# The experimental research on leaders and cooperative behavior

**DOI:** 10.3389/fpsyg.2022.944498

**Published:** 2022-09-23

**Authors:** Xiaogai Fu, Chaoyang Li, Jialin Fu

**Affiliations:** ^1^School of Economics and Management, Zhengzhou University of Light Industry, Zhengzhou, China; ^2^School of Management, Henan University of Technology, Zhengzhou, China

**Keywords:** leaders, public goods game, horizontal reference point, cooperative belief, reciprocity

## Abstract

Leaders are critical to a team or organization, their behavior affects employees’ psychology and their work effort, and then affects the efficiency and innovation of the team or organization. Previous studies have focused on the role model of leaders, ignoring the guiding role of leaders with different efforts. This paper introduces leader decision-making into the game of public goods to investigate the exemplary role of leaders in behavior decision-making. It divides them into three types by setting the investment amount of leaders to explore the mechanism of leaders’ influence in behavior decision-making and behavior change of team members when facing the transformation of leaders with different investment types. This research can provide a significant reference value for enterprises and social organizations on how to play the role of leaders.

## Introduction

Due to the changeable external environment and increasingly fierce competition, the operating mode of team form is gradually popularized (O’Neill and Mclarnon, 2018). However, an unavoidable problem with the team is the free-rider behavior of members. This behavior affects team performance and the psychology and behavior of other employees and eventually results in the overall “inefficiency” of the team. Because when members consider maximizing their private interests, they often ignore the interests of the whole team and even make behaviors that harm the team’s interests. Therefore, how to reduce the free-rider behavior in the team and promote the improvement of team efficiency has become the critical problem that enterprise leaders aim to solve. Besides making decisions on various issues within the team, leaders also need to set an example for the behavior of other organizational members. Hence, they play a crucial role in teamwork.

The role of leaders has always been the focus of academia and industry. Tong (2020) explored the mechanism of its impact on the innovation climate from the perspective of leadership style and defined leadership style as transactional leadership and transformational leadership. There is also literature on the effects of leaders’ negative emotions on employee performance and deviant behavior (Bartels et al., 2022). Research on self-sacrificial leadership has shown that this leadership type can stimulate the identification and trust of members in the organization (Yang et al., 2021), enable employees to cooperate with leaders actively, promote organizational change (Li et al., 2016), make prosocial behavior decisions, and even sacrifice their interests for the organization (Liang and Fan, 2020); Zeng et al. (2020) studied the role of leaders in guiding employees’ decision-making behavior from the perspective of leader role models, and divided leaders into two types: “good” leaders and “bad” leaders. The results showed that the effectiveness of leader role models was minimal because “good” leaders met “bad” followers or “good” followers met “bad” leaders. The above research perspectives on leaders mainly focus on one type, ignoring the impact of leadership type change on employees’ psychology and behavior. Leadership change is also widespread in reality, and there will be significant differences in the impact of different types of leaders on members of the organization. Therefore, it is essential to investigate the effects of different types of leaders and their replacement on organization members.

There are two ways to produce leaders: endogenous and exogenous. Endogenous ways include voluntary endogenous and election endogenous. Exogenous ways mainly include random exogenous and designated exogenous. However, the endogenous leader generation way cannot ensure that leaders can be generated. Every member may be unwilling to play the role of leader under the voluntary endogenous way. The election way may also lead to failure to elect leaders due to the different opinions of team members (Lee et al., 2021). Therefore the leader is generated in an exogenous way in our experiment. Our study tells the subjects that the experimenter will randomly appoint a member as the leader at the beginning of the experiment. The way of appointing exogenous leaders is in line with the Chinese situation. Most leaders are designated by an exogenous superior organization especially in government departments.

In addition to investigating the role model of leaders, this study also divides leaders into three types low, medium and high investors and tries to analyze the investment behavior of employees under the leadership of these three investment types. The subjects were randomly divided into a group of four people in the process of the experiment. One of the members was played by the computer, and the computer decision-making was given the role of leader. The cooperative behavior of members is more out of the social preference of reciprocity or Conditional Cooperation for the two-person group (Fischbacher et al., 2001). The leader often affects and drives the decision-making behavior of other group members through the guiding role of his behavior signal in the behavior decision-making of the four-person group. Therefore, the leaders of this study are closer to the leaders of “self signaling” described by Bénabou and Tirole (2011).

## Theoretical model

A sequential public goods game characterizes the leader’s demonstration behavior in our study. The reason for choosing the public goods game is that leaders’ key task is to promote cooperation among organization members and reduce the free-rider problem in the organization’s management. In addition, this is also the game framework primarily used in the current mainstream literature (Güth et al., 2007; Rivas and Sutter, 2011). The model assumes that an n-person group makes repeated T-period behavioral decisions. Each person is given the initial capital *e* before the beginning of each experimental period. He can choose to invest in public projects of his group or keep them in his private account. In addition, all funds in this period cannot be brought into the experiment of the next period. The investment decision is made with the given initial funds *e* at the beginning of each period of the experiment, which has nothing to do with the capital income of the previous period. If the investment amount of *i* in the group’s public project in *t* period is *g_it_*, the amount of funds retained in the private account is $x_{\mathrm{it}}$, i∈1,2,…,n,t∈1,2,…,T and $x_{\mathrm{it}}+g_{\mathrm{it}}=e$, the total investment in the group’s public project is $\overset{n}{\underset{j=1}{\sum }}g_{jt}$. Assuming that the return on investment coefficient of the group’s public project is $\alpha_{t}$, and $0\leq \alpha_{t}<1<n\alpha_{t}$. Then the payoff of member *i* in *t* period is given by the following function:


$$\pi_{\mathrm{it}}=x_{\mathrm{it}}+\alpha_{t}\left(\overset{n}{\underset{i}{\sum }}g_{{}_{\mathrm{it}}}\right)=e-g_{{}_{\mathrm{it}}}+\alpha_{t}\left(\overset{n}{\underset{j=1}{\sum }}g_{{}_{jt}}\right)\text{.}$$

According to the constraints $0\leq \alpha_{t}<1<n\alpha_{t}$, if the participant is a “rational person” in the sense of economics, the above function has a unique Nash equilibrium solution, i.e., *g_it_* = 0. The solution is also the dominant strategy to maximize the participant’s payoff. However, in terms of maximizing the overall social income, the Nash equilibrium of participants is to invest all the initial funds in the group public projects, that, is *g_it_* = *e* (see the Appendix for the specific derivation process). In this dilemma, individuals are straightforward to take free-rider behavior because maximizing their interests seriously damages the group’s overall and even social benefits.

To avoid collective irrational behavior caused by individual rationality, we try to influence the behavior of other members by allowing leaders to make investment decisions first, and then analyze the investment behavior of subjects under the leadership of different investment types and the change in investment amount of members from meeting “bad” leaders to “good” leaders and from meeting “good” leaders to “bad” leaders. Our study explores the internal mechanism of the leaders’ role to provide an important reference and reference value for enterprises and social organizations.

## Theoretical background and hypotheses development

### Investment and payoff under the influence of leaders

Leaders play a very important role in a family, enterprise or social organization, even the government and international organizations (Lin and Liao, 2020). Leaders play two roles in the market, enterprises and social organizations. One is to provide good or bad information about the project to other organization members in the case of asymmetric information. Generally, there is information asymmetry between leaders and group members, and leaders have private information about investment decisions. Leaders need to use their information advantages to guide the investment behavior of organization members to improve the organization’s overall performance and payoff. For example, Vesterlund (2003) discussed the role of leaders in charitable donations. The research results found that disclosuring the donation amount of leaders with private information could improve the investment amount of others. Andreoni (2006) constructed a dynamic donation game model based on Vesterlund (2003), and believed that leaders with an information advantage could eliminated the information asymmetry among organization members by sending private information to improve the overall investment and performance of the organization.

The second is the self-sacrificing role of the leader in public goods. Leaders influence the decisions of other group members by investing before employees. Huck et al. (2001) conducted 10 periods of random collocation and fixed collocation experiments in a between-group setting; two people were in a group, one subject was a leader and the other was a follower. The leader makes the investment decision first, and the follower invests after seeing the leader’s investment. The results show that the existence of the leader brings more market investment, reduces collaboration, and even improves the overall social welfare; Li et al. (2021a,b) defined the leader as the person who makes decisions first in the process of strategy selection, and believed that the leader-follower model is one of the effective mechanisms that can maintain the order of human cooperation; Nassif et al. (2021) believed that exemplary leadership could stimulate other group members to imitate their behavior and decision-making by taking the lead in investment to stimulate members’ awareness of public cooperation; Van der Heijden and Moxnes (2013) studied the role model of leaders in the bad public goods game framework. The results showed that other members of the organization under the influence of leaders’ decision-making behavior reduced investment in bad public goods projects, the overall cooperation level was improved, and the output of public goods projects similar to environmental pollution was restrained to a certain extent.

Leaders adopt the form of fixed collocation and partnership in this paper, and there is no information asymmetry between leaders and followers. Leaders are divided into three types: low, medium and high through computer play. Middle-type leaders’ investment amount is similar to the average group investment in a non-leader setting. We come to H1 by the above analysis: compared with the benchmark setting, there are significant differences in individual investment and pay0ff between leaders and non-leader settings.

*H1A*: the existence of low investment type leaders reduces the investment and payoff of other group members;

*H1B*: the existence of leaders of medium investment type has no impact on the investment and payoff of other group members;

*H1C*: the presence of high investment type leaders improves the investment and payoff of other group members.

### Individual investment and payoff under the influence of leader investment type and transformation

The investment of leaders will affect the behavior and decision-making of their followers to a certain extent, and then affect the individual investment and payoff and the overall middle-type leaders’ investment and return of the organization. Leadership changes caused by tenure or other reasons are also very common in real enterprises or social organizations, which may be accompanied by the change of leader type. When the leaders in the organization change from low type to high type, the followers will adopt the behavior strategy of “reciprocity” and imitate high type investors to invest more in the projects of their organization according to the positive reciprocity in the reciprocity theory; On the contrary, when the leaders in the organization change from high type to low type, other individuals in the organization will reduce their investment according to the negative reciprocity in the reciprocity theory (Walk, 2022).

Individual investment decisions are affected by the amount of investment of leaders in the presence of leaders and subject to the investment information of themselves and their peers according to the frame of reference theory. Cohn et al. (2014) and Fehr et al. (2021) divided individual reference points into three dimensions when investigating wage reference and employee effort level, namely, vertical reference point, horizontal reference point and current situation reference point. The vertical reference point is based on the salary of the leader (employer), the horizontal reference point is based on the salary of members in organizations with similar situations, and the current reference point is based on the salary standard of the previous period. The results show that the three kinds of reference have an impact on the level of individual effort, and the effect of vertical reference is greater than that of current reference; when studying the influence and mechanism of leaders, the investment of leaders are the vertical reference of individuals, the investment of other members is the horizontal reference of individuals, and their previous investment and payoff are the current reference of individuals. Individuals are affected by these three references at the same time in investment decision-making. Chen et al. (2022) regard the horizontal reference of individuals (innovation activities) as one types of social norm, and found that if market stakeholders such as competitors engaged in extensive innovation activities, the enterprise managers might regard innovation activities as one types of social norm, and thus enhanced the innovation activities of their own enterprises driven by the force of norm compliance. However, individuals pay more attention to the leader’s investment and use it as a reference for investment decision-making under the role of anchoring effect. Therefore, the transformation of the leader’s investment type from low to high is bound to increase the overall investment and payoff of individuals and organizations.

The prospect theory holds that people have a “preconceived” anchoring effect on the objects they contact in advance. At the same time, people’s behavior is situational dependent. The behavior decision-making response under the loss framework is significantly stronger than that under the acquisition framework (Kahneman and Tversky, 1979). The subjects have an anchoring effect on the previous high investment type leaders when the leader’s investment gradually changes from high type to low type. Changing to a lower investment type is a loss for the organization members. The individual responds more strongly to the loss and will be resistant compared with the gain. Thus the negative effect of leaders with low investment is more significant than that of leaders with low investment under the scenario of gradually changing from low investment type to high investment type; on the contrary, the reference point of individuals is the investment of low investment type leaders when leaders gradually change from low to high investment type. The psychology of reciprocity makes them more willing to respond to high investment type leaders and maintain high cooperation when facing high investment type leaders. Thus, there is a significant difference in the amount of investment between the high investment type leader and the low investment type leader.

We propose the following assumptions in view of the above analysis:

*H2A*: the type of leader’s investment is significantly related to individual investment and returns;

*H2B*: low investment type leaders under the two transformation forms have differences in individual investment and returns;

*H2C*: high investment type leaders under the two transformation forms have differences in individual investment and returns.

### Horizontal reference point, cooperation belief and individual investment under different leader types

Individuals are vulnerable to the influence of reference information in the process of investment according to reference theory. Reference information depends on information feedback, and complete information feedback can significantly improve individual investment and individual cooperation level (Irlenbusch and Rilke, 2013). Individual investment is affected by leaders type and restricted by the investment information of peers when he faces the sequential public goods game with leaders (Bahbouhi and Moussa, 2021). The investment information of leaders is the vertical reference of individual decision-making behavior, and the investment information of peers in the previous period is the horizontal reference point for individuals to decide whether to implement cooperative behavior (Cohn et al., 2014; Fehr et al., 2021). These two kinds of references will restrict individual decision-making behavior to a certain extent. The investment of followers will be restrained when the reference point of investment is low. In contrast, the investment of followers will be promoted when the reference point of investment is high.

In addition, Barr (2003) believes that cooperative behavior depends on expected and undesired motivation. He further found that expected motivation has a greater impact on cooperative behavior, and the utility brought by undesired motivation is weak by the residents of 24 villages in Zimbabwe. Fehr (2009) and Sapienza et al. (2013) divided behavioral motivation into belief-based behavior and social preference-based behavior. The belief in belief-based behavior is basically consistent with the expected motivation. The behavior based on social preference is similar to the undesired motivation. Individual behavior decision is affected by the investment information of other group members in the previous period in the public goods game. However, the individual will adjust and form his own cooperative belief after giving this information feedback. The cooperative belief here refers to the individual’s estimate of the average investment of other group members, which is a belief in voluntary cooperation and good faith action. Social norms theory suggests that people voluntarily defend social norms even when their economic interests are not directly affected by norm violations (Yin et al., 2021). Individuals invest in public goods under the influence of their cooperative beliefs which is a social norm, and cooperative beliefs positively affect the voluntary contribution of public goods (Fischbacher and Gächter, 2010).

The following assumptions are put forward based on the above analysis:

*H3A*: the investment of horizontal reference point plays a moderating role between the type of leader and individual investment.

*H3B*: cooperative belief plays a mediating role between the investment of horizontal reference point and individual investment.

### The moderating effect of risk preference between leader type and individual investment

Behavioral economics theory regards risk as an individual’s psychological attitude towards risk, which is an important behavioral basis for making decisions under uncertain conditions (Balafoutas et al., 2012). Due to the obvious differences in the attitudes of decision-makers in risk-taking and dealing with uncertainty, individuals with different risk preferences may give different behavioral decisions on the same decision-making problems (Cadsby et al., 2007). Li et al. (2021b) used loss and gain frameworks to measure risk preference, and found that risk aversion inhibited individual trust behavior, and there was the context-dependence of individual decision-making between them; Davis et al. (2016) used the five-level Likert scale to measure the risk attitude when studying the impact of the heterogeneity of risk preference of senior management team on strategic investment decision-making, and divided it into three types: risk aversion, risk neutrality and risk pursuit, and measured the heterogeneity of team risk preference according to the Blau coefficient (Blau, 1977). The results showed that the heterogeneity of team risk preference was negatively correlated with the job satisfaction of members and positively correlated with decision-making time; Teyssier (2012) believes that risk preference has a negative impact on the voluntary investment decisions of the first decision-makers; Iriberri and Rey-Biel (2019) found that individuals with higher risk preference are more willing to choose variable compensation contracts with relatively higher risk.

Risk preference is situational dependent. Therefore, different risk preferences may lead to significant behavioral differences among organizational members when investigating the impact of leader types on individual cooperative behavior. Individual investment is essentially a risky behavior in the sequential public goods game with leaders. The risk aversion individuals will adopt a conservative strategy and invest fewer funds in organizational projects to maintain a low level of cooperation when the leader’s investment changes from low to high. The risk pursuit preference individuals will contribute a higher amount of investment in investment decision-making and maintain a higher level of cooperation to maximize their long-term interests because the increase of the leader’s investment reduces the uncertainty of risk.

The following assumptions H4 are proposed based on the above analysis:

*H4A*: there is a positive correlation between risk preference and individual investment;

*H4B*: risk preference plays a moderating role between the type of leader’s investment and individual investment.

## Experimental design and process

### Experimental design

Our study uses the sequential public goods game experimental design of Fehr and Gächter (2000) to investigate the impact of leaders and turnover on employee behavior and organizational performance. The specific framework is as follows: each session is composed of 6 4-person groups with 24 participants, of which computers play 6 decision-makers (O’Neill and Mclarnon, 2018). A total of 3 experiments were conducted when investigating the role of leaders, and each experiment was conducted for 10 periods, and the team members adopted the design of partners. The team members and numbers remained unchanged during 10 periods of each experiment (Tong, 2020). A total of 2 experiments were conducted when investigating the impact of different leadership types on employee behavior and organizational performance. Each experiment was conducted for 30 periods and regrouped every 10 periods.

Irlenbusch et al. (2019) adopted exogenous designation to generate leaders, which was in line with the current realistic situation in China. Some studies have also analyzed the difference between electing endogenous and experimenter-appointed exogenous leaders in the environment of asymmetric information. The results show that both of them can better send signals to increase the amount of donations, and the effect of the third-party appointed exogenous leader mechanism is better (Potters et al., 2007). Rivas and Sutter (2011) found the opposite conclusion that the election of endogenous leaders is better than external leaders in improving organizational donations. Güth et al. (2007) found that election endogenous and random exogenous have good effects in improving group investment. Arbak and Villeval (2013) found that leaders generated by voluntary endogenous can improve the overall investment of the organization, but this method has the disadvantage of “leader dystocia.”

The above research found that leaders generated by either endogenous or exogenous methods can effectively improve the overall investment level of the organization compared with the situation where there is no leader. In addition, some studies pay attention to the exemplary role of leaders in bad public goods, such as environmental pollution. The results show that the existence of leaders significantly reduces the overall cooperation level of the organization. We use the random exogenous method to generate leaders, and the leaders are played by computers. The advantage of choosing a computer as the leader is that we can clearly distinguish the types of leaders and better separate the guiding role of different types of leaders on the behavior of organizational members and organizational performance. We told the subjects that leaders were randomly assigned in order to reflect the authenticity of leaders during the experiment.

There are 6 experimental settings in this study. In addition to 16 participants in the benchmark setting, 18 participants in other settings. One experiment is conducted in each setting, with 116 participants. The subjects were all freshmen to junior students of a university, with an average age of 22. The subjects were selected through the questionnaire and conducted gender balance. The specific settings are shown in Table 1.

**Table 1:** Experiment setup and type.

Experimental setup	Leader exist	Leader investment type	Number of teams	Actual number of participants
T1	No	–	4	16
T2	Yes	Low	3	18
T3	Yes	Medium	3	18
T4	Yes	High	3	18
T5	Yes	Low → medium → high	3	18
T6	Yes	High → medium → low	3	18

The number of groups is 4 in the six settings. Since the leaders in T2, T3, T4, T5, and T6 are all played by computers, the number of groups here excludes the leaders played by computers, so it is 3; similarly, there are 6 groups in T2, T3, T4, T5, and T6. When counting the real number of participants, excluding 6 leaders played by computers, it is 18.

#### Benchmark setting

This setting is a public goods experiment of 20 periods without the leader. Each experiment has 16 subjects, and each group has 4 people, and the grouping and member number remained unchanged throughout the experiment. Before the beginning of each period of the experiment, each subject is given an initial capital of 50G$ to invest in the group project. The payoff function of subject i in the group is determined by $\underset{i}{\prod }=50-x_{i}+0.5\overset{4}{\underset{j=1}{\sum }}x_{j}$. Where $x_{i}$ represents the amount of investment that member i has invested in the group project, and $\overset{4}{\underset{j=1}{\sum }}x_{j}$ represents the total amount of investment that member n have made in the group project. The funds are retained by the individual belong to themselves. The funds invested in the group project are halved, but the individual can share the investment of other group members in the group project. We can better investigate the decision-making behavior and cooperation level of individuals through this setting in the case of conflict between their interests and overall interests.

#### Experimental setup under low investment type leaders

The payoff function of members in this setting is similar to the benchmark setting. What is different from the benchmark setting is the existence of leaders. Each experiment has 18 subjects participated and each group of 3 people conducted 10 periods of sequential public goods game. The member number and grouping remain unchanged during the whole experiment. This setting is more in line with the actual situation of the enterprise, and the conclusions are more valuable for reference. In addition, each group also has a low investment leader played by a computer. The leader is a “pioneer” in the public goods game. It is necessary to randomly select an integer from 3-5G$ as its investment in the group project. After seeing the leader’s investment, the other three group members will make investment decisions. This setting can better examine group members’ behavioral decision-making rules and cooperation levels when facing leaders with low investment.

#### Experimental setup under medium investment type leaders

This setting is the same as setting (Tong, 2020), only difference is the investment amount of the virtual leader. The investment amount of the leader of the medium investment type in the group project is randomly selected from 20 to 22G$. This setting is to be consistent with the investment amount of the benchmark setting. The research shows that people’s investment in public goods generally accounts for about 40% of the initial resource endowment in reality (Ibanez and Schaffland, 2018). Therefore, the investment amount of leaders with medium is consistent with that without leaders, so as to compare it to the benchmark setting and the difference between leaders with low investment amount and leaders with high investment amount.

#### Experimental setup under high investment type leaders

This setting is consistent with settings (Tong, 2020) and (Bartels et al., 2022) except for the investment of leaders. We set the leader’s investment in the group project to be randomly selected from 42 to 45G$ to reflect the power of the leader’s role model. Frackenpohl et al. (2016) defined good leaders as leaders whose investment is close to all initial funds, and bad leaders as first decision makers whose investment is zero when studying collective leaders and individual leaders. In view of this, we set leaders with an investment of 42–45G$ as high investment leaders, and investigate their role model among group members and their impact on the level of group cooperation.

#### Experimental setup of low, medium and high investment type leaders

Under the background of the Chinese system, the conflict between major shareholders and management has always been the focus of attention. Cheng et al. (2020) believe that there is a positive correlation between the occupation of funds by major shareholders and the change of management personnel. Such occupation has an adverse impact on the development of enterprises. In fact, in addition to many factors affecting the change of leadership, the change of leadership will also impact employees’ psychology, and then affect employees’ cooperation level and organizational performance. Therefore, we try to study the inhibition or promotion of leader type on organizational member behavior and organizational performance through the change of leader type. The experiment set up 30 periods, regrouping every 10 periods, and changing the investment type of leaders. The first 10 periods are low investment type leaders, the middle 10 periods are medium investment type leaders, and the last 10 periods are high investment type leaders. The experimental setup can better investigate the impact of the change of leadership investment type from low to high on team members’ decision-making behavior and organizational performance.

#### Experimental setup of high, medium and low investment type leaders

The order of leaders’ investment types in this setting is the opposite of that in setting (Li et al., 2016). The first 10 of the 30 periods are high investment leaders, the middle 10 periods are medium investment leaders, and the last 10 periods are low investment leaders. This experimental setup investigates the behavior change of organization members when the organization gradually changes from high investment leader to low investment leader.

### Experimental process

Six experiments were set up in the laboratory of School of Management of a University from December 2017 to January 2018, and 116 college students participated in the experiment. The decision-making experiment includes two parts: computer decision-making and questionnaire survey. The programs of these two parts are realized with the help of z-Tree software (Fischbacher, 2007). Each experiment lasted about 60 min, and the average payoff of the subjects was 25 yuan.

The whole experimental process mainly includes five stages:

#### Plane arrangement stage before experiment

The subjects were recruited through the questionnaire star. After the subjects arrive at the laboratory and sign in, the experimental assistant will lead them to the corresponding experimental seat to avoid the subjects choosing the seat according to their preferences and interests. The experimental assistant arranges the seats for the subjects according to gender, major and college. Every two subjects are separated by two seats to ensure that the subjects do not know each other and avoid communication. At the same time, the subjects did not know their number and grouping in advance. They were only informed in the computer experiment stage to ensure the “anonymity” of the whole experimental process.

#### Understanding stage of experimental instructions

After all the subjects arrived, the experiment officially began. The experimental assistant will distribute the experimental instructions to each subject and give them 5 min of self-reading time. Then the experiment host explains the experiment description and answers questions privately to ensure that the subjects accurately understand the experiment description. In addition, in order to test whether the subjects really master the whole decision-making process, they also need to correctly complete the pre-designed test questions including yes/no judgment questions and blank filling questions. After the test questions are correctly completed, the experiment host will briefly answer the questions existing in the test process and explain the interface content in the process of computer experiment to avoid the delay of time or arbitrary decision-making due to the unfamiliar of the interface or misunderstood in the experimental process.

#### Economic decision-making stage

The economic decision-making stage and the questionnaire survey stage are collectively referred to as the computer decision-making stage. Subjects were divided into groups before making economic decisions (O’Neill and Mclarnon, 2018). The benchmark experiment setting requires the subjects to make investment decisions on their group project, and the investment amount is an integer of 0–50G$; Then, the subjects need to estimate the average investment of the other three group members (Tong, 2020). In the non-benchmark experiment setting stage, the first person in the group makes investment decisions, other subjects make investment decisions after seeing the investment amount of the first person and estimate the average investment of the other two members except the first person. After the investment decision interface is submitted, you can enter the estimation interface. The setting of investment decision before estimation avoids the possible influence of the estimated value of the investment decision (Irlenbusch et al., 2019). Information feedback interface appears after the investment and estimation decision is completed. This interface displays individual number, investment amount, payoff information, the group average investment amount, estimated value, and real value, as well as the number, investment amount and payoff information about other group members.

Information feedback draws on the personal information feedback of Sell and Wilson (1991) and adopts “partner design” (Weimann, 1994; Bigoni and Suetens, 2012; Irlenbusch and Rilke, 2013), that is, setting (O’Neill and Mclarnon, 2018; Yang et al., 2021) keep the grouping and individual number unchanged throughout the experiment, and setting (Li et al., 2016; Liang and Fan, 2020) regroup every 10 periods. By comparing setting (Tong, 2020; Yang et al., 2021; Bartels et al., 2022) with setting (O’Neill and Mclarnon, 2018), our study analyzes the impact of leader type on individual and group investment, and analyses the mechanism of leader type change on individual investment level and overall group performance by comparing setting (Li et al., 2016; Liang and Fan, 2020).

#### Questionnaire survey stage

Economic decision-making is followed by the questionnaire stage, which mainly includes two parts. The first part is the investigation of basic personal information, including gender, age, major, native place, family income, parents’ educational background, whether they are the only child, whether they come from rural or urban areas, whether they have educational experience in economics and whether they understand game theory. The family income is in the form of a seven-level Likert scale, with asking the subjects “what do you think your family income is _____ (between 1 and 7, of which 1 represents very poor and 7 represents very rich)”; Parents’ educational background is in the form of multiple-choice questions with the form of six-level Likert scale. The options are “primary school and below, junior middle school, senior high school, junior college, undergraduate and master’s degree or above.”

Kurzban and Houser (2005) believes that the subjects in the public goods experiment include three types: conditional collaborators, unconditional collaborators and free riders. Repeated experiments found that unconditional collaborators invest more in group projects than conditional collaborators, and free riders have the lowest average investment in group projects among the three types. Conditional collaborators and conditional cooperation behaviors exist widely in enterprises and social organizations (Fischbacher et al., 2001; Kurzban and Houser, 2005). Fischbacher et al. (2001) first studied the problem of conditional cooperation and defined conditional cooperation as the increase of individual investment with the increase of others’ investment. Individuals need to choose cooperative decision-making according to the cooperative behavior of others. The results showed that 50% of the subjects were conditional collaborators. Fischbacher and Gächter (2010) further found that although most of the subjects are conditional collaborators, they have certain “self-partiality” characteristics and they are not perfect conditional collaborators. Most conditional collaborators’ investment in the group will be slightly less than the average investment of other members of the group; In addition, other studies have found that the investment amount of conditional collaborators is affected by the expected and actual value of the average investment amount of other members of the group, and there is a significant positive correlation (Croson, 2007).

Therefore, we also tested the subjects’ altruistic preference, cooperative belief and risk preference in the second part. Altruistic preference is to ask the participants to answer “suppose you and any one of the other participants form a group and jointly allocate 100G$. It is up to you to decide how much to give to the other participant, and the rest is left to yourself, and the other participant can only accept it. So, how much do you decide to give to the other participant?” Cooperation belief is an individual’s expectation of the average investment amount of other members of the group. The measurement of this variable is carried out in the economic decision-making stage. After the subjects invest in the group project, let the subjects answer “please estimate the average investment amount of the other three members of your group (fill in the integer from 0 to 50),” and the question becomes “please estimate the average investment amount of the other two members of your group except the leader (fill in the integer from 0 to 50)” in the leader settings (Fischbacher and Gächter, 2010; Dufwenberg et al., 2011). The measurement of risk attitude is mainly in the form of a seven-level Likert scale by asking the subjects “please give the degree of risk you are willing to take (choose between 1 and 7, 1 means very dislike and 7 means very like).”

#### Payoff payment and interview stage

When filling in the questionnaire, the experiment host randomly selected any one of the 10 periods and converted it into cash in the proportion of 4:1 as the experimental payoff to the subjects. Remind the subjects to remember their personal number during the experiment, the subjects were paid privately according to their personal numbers. Afterward, 3–4 subjects were randomly selected for post-experiment interviews to ask about how to make decisions and suggestions on the experiment to ensure that they fully understand the experimental process and make serious decisions.

## Analysis of experimental results

### Descriptive statistics and *t*-test analysis

#### Overall feature analysis

There were 75 females and 41 males in the whole experiment, and females accounted for about 64.7%, only children accounted for 27%, cities accounted for 30, and 87% of the subjects had economic learning experience. The educational background of fathers is slightly higher than that of mothers (2.34 *vs*. 2.16). The educational background of fathers is concentrated in junior middle school and senior high school, accounting for 67% of the total, and the educational background of mothers is concentrated in primary school and junior high school, accounting for 72% of the total; The mean value of altruistic preference is 44; The risk preference measured by the seven-level Likert scale is concentrated in the values of 3 and 4, indicating that most subjects are risk neutral.

#### Individual investment and payoff under different settings

Table 2 shows individuals’ investment amount and payoff under the six settings. Individual investment under T4 (high leader type) is slightly higher than that under T1 (no leader). The average individual investment in other settings is less than that in the experimental setting without a leader. The existence of low and medium investment type leaders reduces the investment of other group members. Only the high investment type leader setting slightly increased the investment of other group members; In terms of individual payoff, setting T4 is the highest, which is 80.461, and setting T2 is the lowest, which is 56.179. Settings 3 and 1 decrease slightly (70.775 *vs*. 66.533), and the income under settings T2 and T3 is relatively concentrated (the standard deviation is 8.611 and 10.070). Through data analysis of investment amount and payoff, H1A and H1C are basically verified, and H1B is not verified.

**Table 2:** The investment amount and payoff of individuals under the six settings.

Experimental setup	Sample size	Average investment		Average payoff	
		Mean value	Standard deviation	Mean value	Standard deviation
T1	320	20.775	17.464	70.775	17.764
T2	180	8.858	11.757	56.179	8.611
T3	180	14.106	12.151	66.533	10.070
T4	180	22.022	17.985	80.461	13.217
T5	540	13.917	14.866	67.725	14.663
T6	540	12.741	15.081	67.137	15.237

To master the overall investment and payoff, the average investment and payoff in the table removed the investment of the leaders in settings T5 and T6.

Table 3 shows the *t*-test results of investment amount and payoff by setting T2, T3, T4, and T1, respectively. There is no difference between T4 and T1 (*t*-value is −0.806, *p*-value is 0.421) and there are significant differences between other settings and T1 In the *t*-test of investment amount. There is a significant difference between T1 and T2 in the *t*-test of payoff. The investment amount of H1C has not been verified, other assumptions of H1 have been verified.

**Table 3:** The *t*-test of individual investment and payoff.

		T1 *vs*. T2	T1 *vs*. T3	T1 *vs*. T4	T5l *vs*. T6l	T5 m *vs*. T6 m	T5 h *vs*. T6 h
Investment	*T*	8.277	6.705	−0.806	3.323	3.126	−2.196
	*p*	0.000	0.000	0.421	0.001	0.002	0.029
Payoff	*T*	10.475	3.821	−7.223	2.057	1.870	−1.490
	*p*	0.000	0.000	0.000	0.041	0.062	0.137

T1 to T6 represent 6 settings, of which t5l represents 10 periods of experiments in which the leader type in setting 5 is low investment type, T5 m represents 10 periods of experiments in which the leader in setting 5 is medium investment type, and t5 h represents 10 periods of experiments in which the leader in setting 5 is high investment type. T6l, t6 m, and t6 h are similar to setting T5.

#### Individual investment amount and payoff under the influence of leader investment type transformation

It can be seen from Table 4 that when the leader’s investment type is in ascending order (T5), the average value of individual investment increases from 8.716 to 15.083 and then to 17.95, and the average value of payoff increases from 56.108 to 67.642 and then to 79.425, all of which maintain an upward trend. The leader’s investment type is positively correlated with individual investment and payoff. A similar situation was found in T6. The individual investment decreased from 22.094 to 11.128 and then to 5, and the payoff decreased from 81.497 to 65.664 and then to 54.25 when the leader’s investment type appeared in descending order, which maintained a downward trend as a whole. It is found that the type of leader investment is positively correlated with individual investment and payoff through the data analysis of T5 and T6, which basically verifies H2A.

**Table 4:** The leader type transformation and individual investment and payoff.

Experimental setup	Sample size	Average investment		Average payoff	
		Mean value	Standard deviation	Mean value	Standard deviation
T5d	180	8.716	11.695	56.108	9.206
T5z	180	15.083	12.608	67.642	10.192
T5g	180	17.95	18.022	79.425	13.618
T6d	180	5	9.401	54.25	7.888
T6z	180	11.128	11.370	65.664	9.877
T6g	180	22.094	17.786	81.497	12.758

Comparing the individual investment amount and payoff of leaders with low investment amount in T5 and T6, it is found that the individual investment amount and payoff under the ascending order of leader investment amount (T5) are 8.716 and 56.108 respectively, and the individual investment amount and payoff under the descending order (T6) are 5 and 54.25, respectively. The individual investment amount and payoff guided by leaders with low investment amount under the ascending order are higher than those in the descending order. The *t*-test of the individual investment amount and payoff of the two settings shows that the value of *p* of the investment *t*-test is 0.001 and the value of *p* of the payoff *t*-test is 0.041, which are significant. There are significant differences between the investment amount and payoff, which basically verifies H2B.

Comparing the individual investment amount and payoff of leaders with high investment amount in T5 and T6, it is found that the individual investment amount and payoff of leaders with high investment amount in ascending order (T5) are 17.95 and 79.425, respectively, and the individual investment amount and payoff of leaders with high investment amount in descending order (T6) are 22.092 and 81.497, respectively. The individual investment amount and payoff guided by leaders with high investment amount in ascending order are lower than those in descending order; The t-test of the individual investment amount and payoff of the two settings shows that the value of *p* of the investment amount and payoff *t*-test is 0.029 and the value of *p* of the payoff *t*-test is 0.137. There is a significant difference in the investment amount and no significant difference in the payoff. In H2C, the investment part is verified, while the payoff part is not verified.

### Regression analysis

#### Individual investment and payoff under the influence of leaders

To further test the mechanism of the existence of leaders and the type of leadership investment on individual investment, the next step is to analyze it by a regression model. The independent variables in Table 5 are the amount of individual investment in each period, and the independent variables variable setting (treat) is a dummy variable. Models 1-1 and 1-2 are the regression between leaders with no leader and leaders with low investment, no leader takes 0, and leaders with low investment take 1; Models 2-1 and 2-2 are regression under the existence of no leader and medium investment type leader. No leader takes 0 and medium investment type leader takes 1; Models 3-1 and 3-2 are regression with no leader and high investment type leader. No leader takes 0 and high type leader takes 1. The control variables were gender (Gender), altruistic preference (altruistic), number of periods (period), economic study experience (economic), family income (income), parental education (father × mother) and single child (single). The results show that the existence of low investment type leaders and medium investment type leaders significantly reduces the amount of individual investment, and the existence of high investment type leaders significantly improves the amount of individual investment. The amount of individual investment in H1A and H1C is verified. In H1B, although there is a significant difference between the amount of individual investment in the presence of medium investment type leaders and that without leaders, it significantly reduces the amount of individual investment.

**Table 5:** Individual investment with or without leaders.

Independent variables	No leader *vs*. low type leader		No leader *vs*. medium type leader		No leader *vs*. high type leader	
	Model 1-1	Model 1-2	Model 2-1	Model 2-2	Model 3-1	Model 3-2
Treat	−12.058*** (−8.28)	−12.022*** (−7.17)	−7.669*** (−6.70)	−7.477*** (−5.64)	1.319* (0.81)	4.938*** (2.57)
Gender		1.253 (0.82)		−0.305 (−0.26)		2.068 (1.25)
Altruistic		0.169*** (3.65)		0.170*** (4.52)		0.078 (1.22)
Period		0.528*** (3.93)		0.219* (1.80)		0.520*** (3.42)
Economic		−6.413** (−2.09)		−5.078** (−2.20)		4.885 (0.96)
Income		1.189* (1.76)		0.871 (1.53)		2.045** (2.46)
Father × mother		0.168 (1.20)		−0.027 (−0.23)		0.401* (1.72)
Single		−6.896*** (−3.35)		−6.628*** (−4.51)		−11.250*** (−4.54)
*N*	500	500	500	500	500	500
*R* ^2^	0.121	0.223	0.062	0.150	0.001	0.108

*means significant at the level of 10%, **means significant at the level of 5%, ***means significant at the level of 1%. *T* value in parentheses. *N* represents the sample size.

When investigating the influence mechanism of the existence of leaders on individual payoff, we take the individual payoff in each period as the dependent variables. The independent variables and control variables are the same as above. The results are shown in Table 6. The existence of low investment type and medium investment type leaders reduces the individual payoff, while the existence of high investment type leaders improves the individual payoff. H1A and H1C were verified, and H1B was not verified, but it is found that it is significantly different from the leaderless setting.

**Table 6:** Individual payoff with or without leaders.

	Model 1-1	Model 1-2	Model 2-1	Model 2-2	Model 3-1	Model 3-2
Treat	−14.667*** (−10.48)	−12.225*** (−7.32)	−4.122*** (−3.82)	−2.700** (−2.08)	10.722*** (7.22)	12.065*** (6.80)
Gender		−0.460 (−0.30)		0.225 (0.19)		3.094** (2.03)
Altruistic		0.002 (0.04)		0.012 (0.31)		−0.004 (−0.07)
Period		0.568*** (4.24)		0.380*** (3.18)		0.566*** (4.03)
Economic		0.132 (0.04)		−2.095 (−0.93)		−3.838 (−0.82)
Income		1.212* (1.80)		0.589 (1.06)		2.533** (3.30)
Father × mother		−0.144 (−1.03)		−0.028 (−0.24)		−0.156 (−0.72)
Single		−2.001 (−0.98)		−1.980 (−1.38)		−2.728*** (−1.19)
*N*	500	500	500	500	500	500
*R* ^2^	0.181	0.222	0.021	0.042	0.095	0.159

Treat is taken as 0 in benchmark setting. The leader of low investment type is taken as 1 in Model 1-1 and 1-2, while the leader of medium investment type is taken as 1 and the leader of high investment type is taken as 1, respectively, in Model 2-1 and 2-1 and Model 3-1 and 3-2. *means signifcant at the level of 10%, **means signifcant at the level of 5%, ***means signifcant at the level of 1%. *T* value in parentheses. *N* represents the sample size.

#### The influence mechanism of leader’s investment type and transformation on individual investment and payoff

To further analyze the relationship between the type of leader investment and individual investment and payoff, we use the method of linear regression analysis. Gender (Gender), altruistic preference (altruistic), number of periods (period), economic education experience (economic), family income (income), parental education (father × mother) and single child (single) are the control variables, individual investment and payoff are the independent variables, and the type of leader investment is the dependent variable. The type of leader with low investment is 0, take 1 for medium investment type and 2 for high investment type. The results show that the type of leader’s investment is significantly positively correlated with individual investment and payoff. The correlation coefficients of individual investment and payoff are 6.582 and 12.641, values of *p* are all 0.000. H2A is verified.

When investigating the impact of the transformation of leader investment type on individual investment and payoff, our study selects the three types of low, medium and high investment in setting T5 to match setting T6 respectively, and tests them by stepwise regression. The results are shown in Tables 7, 8. Model 1-1 and 1-2 is the matching of low investment type leaders under the two settings, and model 1-2 adds a series of control variables on the basis of Model 1-1, which are the same as those analyzed above. Models 2-1 and 2-2 and Models 3-1 and 3-2 matches the leaders of medium investment type and high investment type, respectively. The results show that the existence of low investment type leaders reduces the individual’s investment and payoff, and the *t*-test results are significant. The *t*-value of investment *t*-test is 3.323, value of *p* is 0.001, and the *t*-value of payoff *t*-test is 2.057, value of *p* is 0.041. Support H2B. In addition, it is also found that the individual investment and payoff in the ascending investment type are greater than those in the descending order in the regression. The existence of high investment type leaders with reference is not as good as the individual investment without reference. The individual investment of high investment type leaders in ascending order is lower than that in descending order, and the t-test is significant, but the *t*-test and regression results of individual payoff are not significant, the transformation of leader investment type cannot significantly affect individual payoff. In H2C, the investment part is verified, while the payoff part is not verified.

**Table 7:** Individual investment under the influence of leader’s investment type order.

Independent variables	Low type leader		Medium type leader		High type leader	
	Model 1-1	Model 1-2	Model 2-1	Model 2-2	Model 3-1	Model 3-2
Treat	−3.717*** (−3.32)	−3.554*** (−3.01)	−3.956*** (−3.13)	−2.372* (−1.89)	4.144** (2.20)	5.985*** (3.16)
Gender		0.215 (0.17)		−0.068 (−0.05)		0.389 (0.19)
Altruistic		−0.021 (−0.63)		0.079** (2.21)		0.112** (2.06)
Period		−0.819*** (−4.32)		−1.463*** (−7.29)		−1.313*** (−4.33)
Economic		−0.438 (−0.24)		−4.826** (−2.45)		−1.465 (−0.49)
Income		1.310** (2.13)		1.727*** (2.65)		6.221*** (6.33)
Father × mother		0.033 (0.32)		−0.234** (−2.11)		−0.415** (−2.48)
Single		−0.310 (−0.22)		−5.244*** (−3.52)		−5.675** (−2.52)
*N*	360	360	360	360	360	360
*R* ^2^	0.030	0.100	0.027	0.072	0.013	0.176

*means significant at the level of 10%, **means significant at the level of 5%, ***means significant at the level of 1%. *T* value in parentheses. *N* represents the sample size.

**Table 8:** Individual payoff under the influence of leader’s investment type order.

Independent variables	Low type leader		Medium type leader		High type leader	
	Model 1-1	Model 1-2	Model 2-1	Model 2-2	Model 3-1	Model 3-2
Treat	−1.858** (−2.06)	−1.190* (−1.22)	−1.978* (−1.87)	−1.883* (−1.68)	2.072 (1.49)	2.626* (1.80)
Gender		−1.764* (−1.69)		−0.700 (−0.58)		0.288 (0.18)
Altruistic		0.018 (0.65)		−0.028 (−0.87)		−0.106** (−2.54)
Period		−0.394** (−2.52)		−0.725*** (−4.03)		−0.629*** (−2.70)
Economic		0.521 (0.34)		−0.306 (−0.17)		−2.801 (−1.23)
Income		−0.368 (−0.72)		−1.186** (−2.03)		3.035*** (4.01)
Father × mother	360	0.114 (1.31)		0.194* (1.95)		−0.107 (−0.83)
Single		−0.521 (−0.45)		0.156 (0.12)		−4.862*** (−2.81)
*N*		360	360	360	360	360
*R* ^2^	0.012	0.042	0.010	0.072	0.006	0.097

*means significant at the level of 10%, **means significant at the level of 5%, ***means significant at the level of 1%. *T* value in parentheses. *N* represents the sample size.

#### Horizontal reference point of investment, risk preference and individual investment

**Figure 1:**
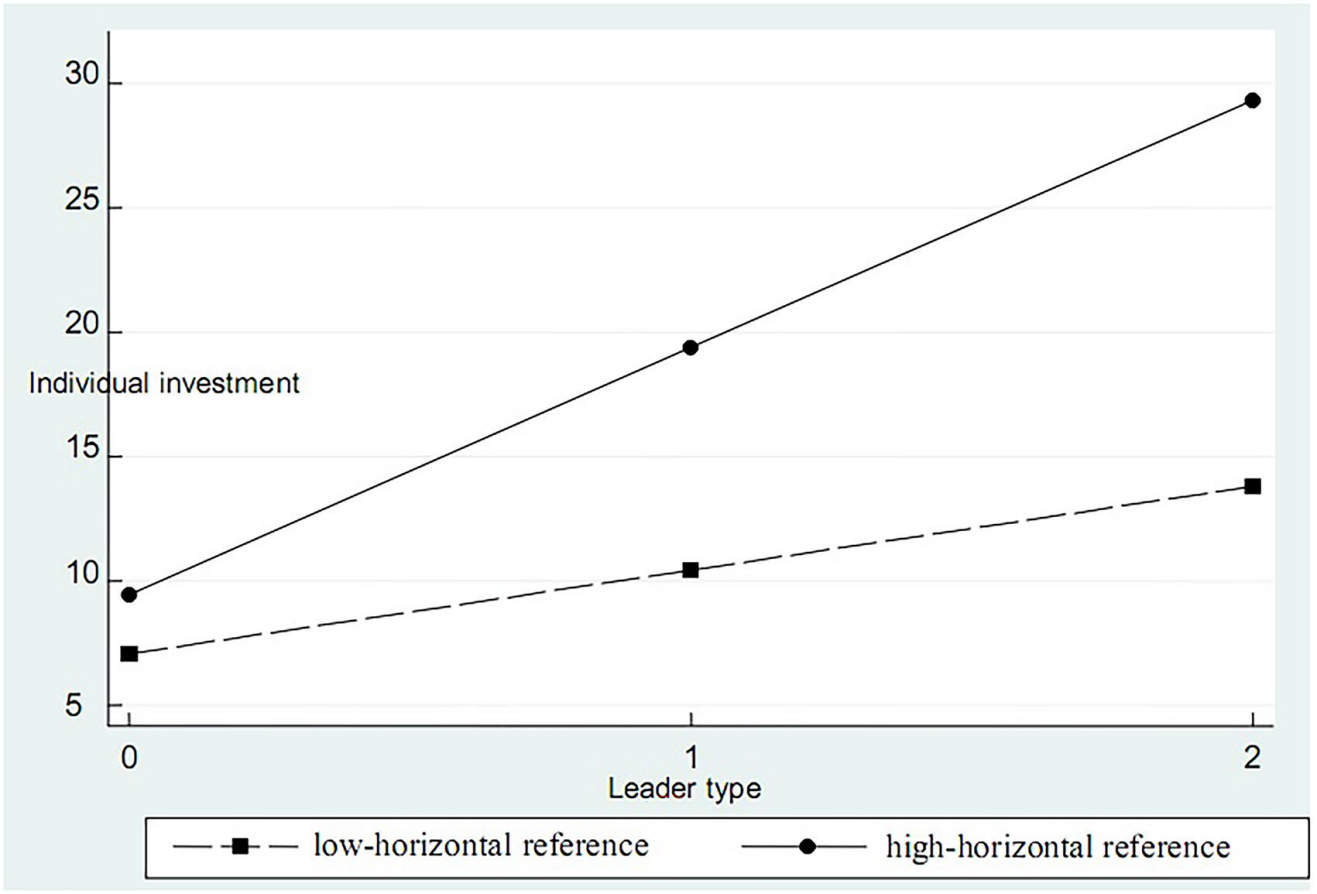
Moderator effect of horizontal reference point.

**Figure 2:**
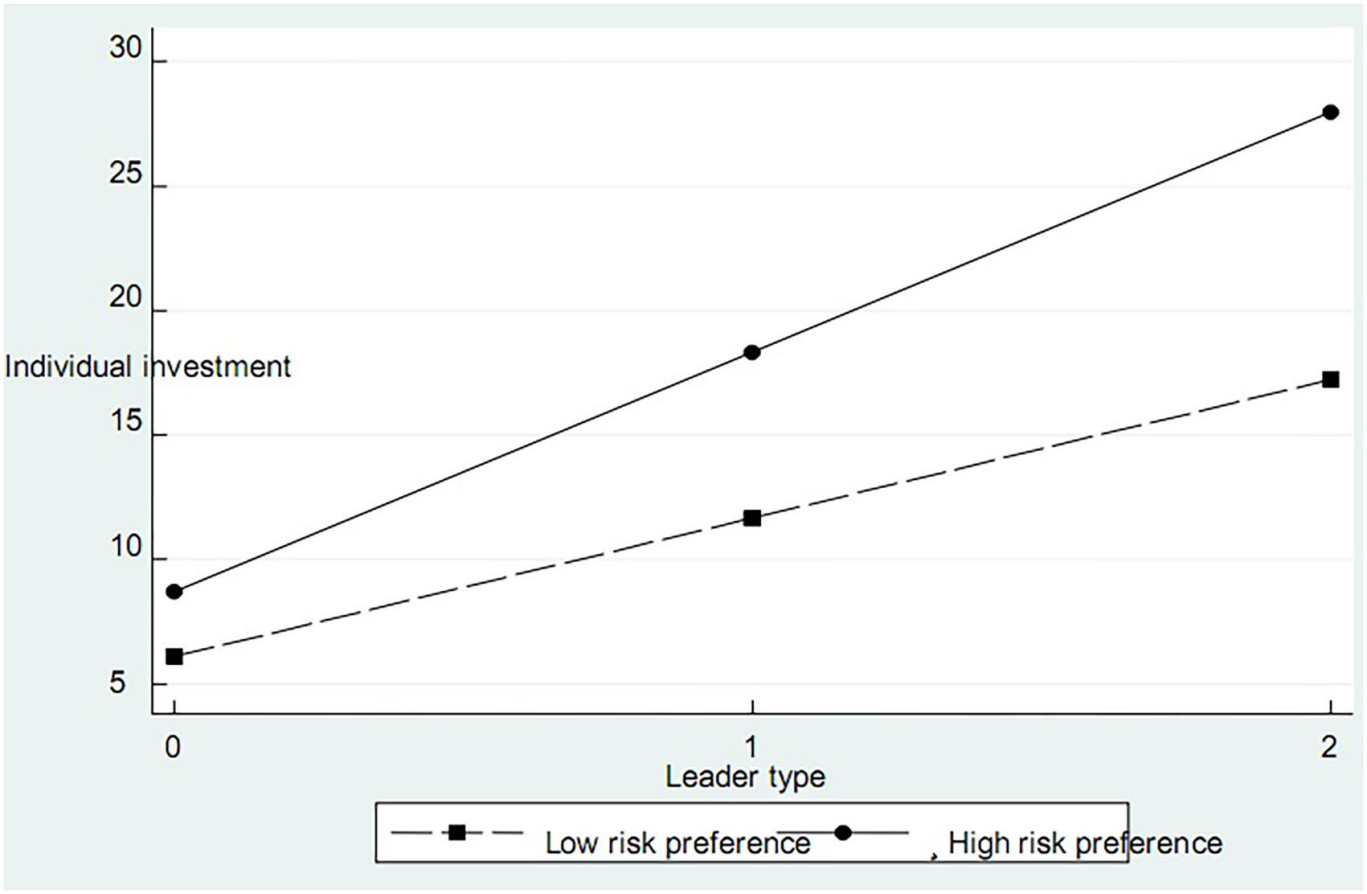
Moderator effect of risk preference.

In order to further analyze the role of leaders’ existence and their investment types in individual investment, we take the lag of the average investment amount of other group members and risk preference as moderator variables and test their role between leaders’ investment types and individual investment in stepwise regression. See Table 9 for details. According to the classification of reference standards by Cohn et al. (2014) and Fehr et al. (2021), we define the lag of the average investment amount of other group members as the horizontal reference point of investment, and risk preference is measured using a seven-level Likert scales. According to M2-1 and 2-2 in Table 9, there is a positive correlation in risk preference and individual investment with leaders (coefficients are 1.950 and 1.338, respectively), supporting H4A. It can be seen from M1-1 and 1-2 in Table 9 that the horizontal reference point plays a positive regulatory role between the leader’s investment type and the individual’s investment. The larger the investment horizontal reference point, the more the individual’s investment. The investment horizontal reference point is divided into low-horizontal reference investment and high-horizontal reference investment according to the average value of the investment (see Figure 1). The results show that high horizontal reference investment has a greater impact on the relationship between leader investment type and individual investment, which supports H3A. According to M3-1 and 3-2 in Table 9, risk preference plays a positive regulatory role between the type of leader’s investment and individual investment. Further, the risk preference is divided into low-risk preference and high-risk preference according to whether the value of risk preference is greater than 3 (see Figure 2). The results show that individuals with high risk preference increase the amount of individual investment faster with the increase of the type of leadership investment in the relationship between the type of leader’s investment and individual investment, and the H4B is verified.

**Table 9:** Horizontal reference point of investment, risk preference and individual investment with leaders.

Independent variables	Horizontal reference lag		Risk preference			
	M1-1	M1-2	M2-1	M2-2	M3-1	M3-2
Type	1.384* (1.89)	1.881*** (2.59)		−1.883* (−1.68)	2.495 (1.64)	2.495* (1.73)
avg(1)	0.374*** (5.41)	0.293*** (4.20)				
Risk			1.950*** (4.61)	1.338*** (2.90)	0.575 (0.92)	−0.037 (−0.06)
avg(1) × type	0.113*** (2.65)	0.115*** (2.73)				
Type × risk					1.375*** (2.85)	1.375*** (3.01)
Gender		0.569 (0.64)		0.571 (0.58)		0.571 (0.63)
Altruistic		0.072*** (2.98)		0.062** (2.33)		0.062** (2.52)
Period		−0.763*** (−4.83)		−1.198*** (−7.96)		−1.198*** (−8.64)
Economic		−1.623 (−1.21)		−1.983 (−1.35)		−1.983 (−1.46)
Income		1.767*** (3.90)		2.677*** (5.28)		2.677*** (5.72)
Father × mother		−0.166** (−2.20)		−0.256*** (−3.03)		−0.256*** (−3.29)
Single		−1.919* (−1.91)		−2.888** (−2.55)		−2.888*** (−2.77)
*N*	972	972	1,080	1,080	1,080	1,080
*R* ^2^	0.285	0.042	0.019	0.107	0.155	0.243

*means significant at the level of 10%, **means significant at the level of 5%, ***means significant at the level of 1%. *T* value in parentheses. *N* represents the sample size.

#### Horizontal reference point of investment, cooperative belief and individual investment

**Figure 3:**
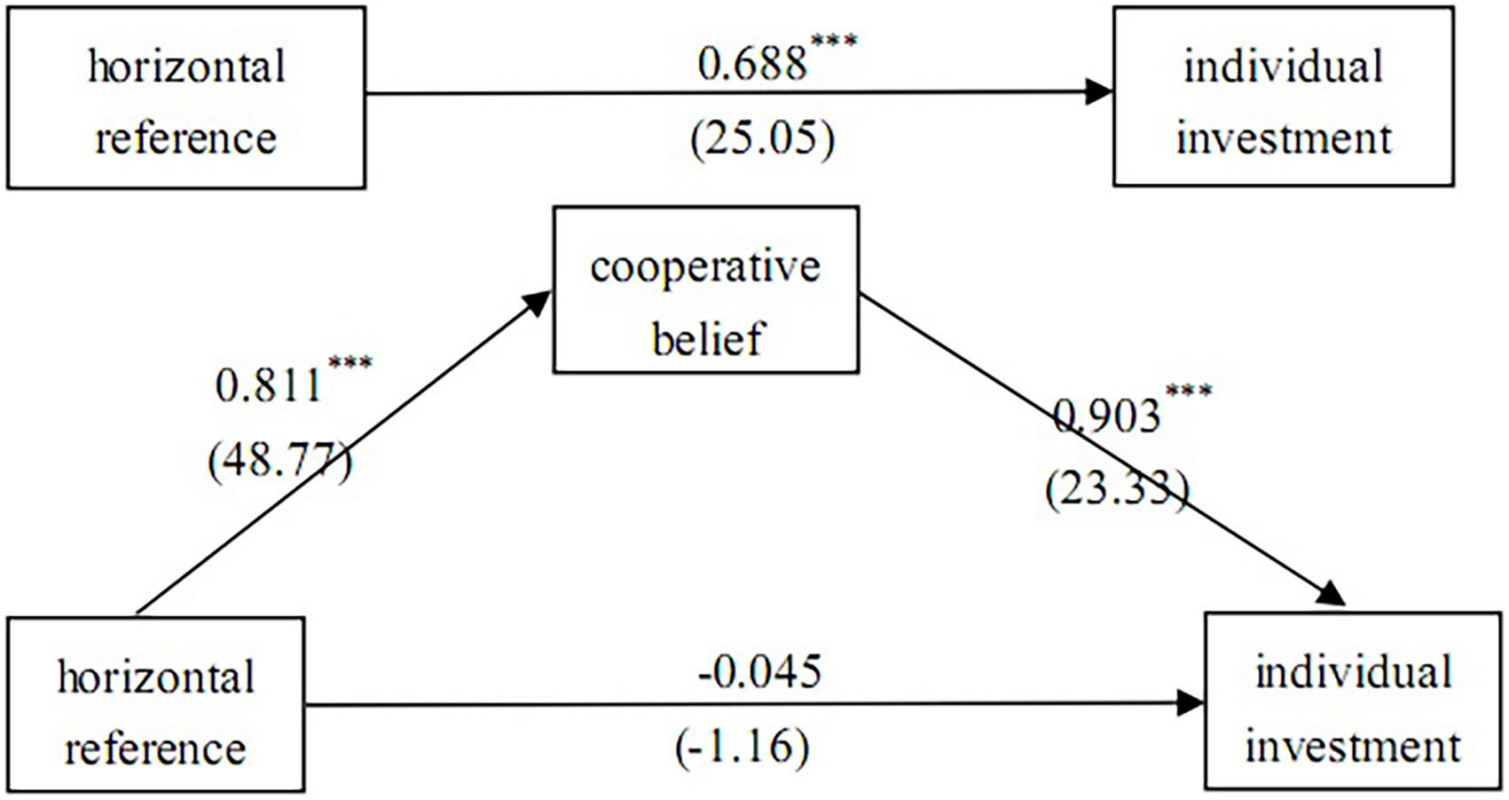
Mediation test results of horizontal reference point of investment-cooperative belief-individual investment.

The intermediary effect test of cooperative belief draws lessons from the analysis methods of Preacher and Kelley (2011), and the results of path analysis are shown in Table 10 and Figure 3 (O’Neill and Mclarnon, 2018). The horizontal reference point of investment (independent variable) significantly affects the individual’s cooperation belief (intermediary variable; regression coefficient is 0.811, *t* value is 48.77); (2) the horizontal reference point of investment (independent variable) significantly affects individual investment (dependent variable; regression coefficient is 0.688, *t* value is 25.05; Bartels et al., 2022). When considering the horizontal reference point of investment and cooperation belief, it is found that the correlation between individual investment and the horizontal reference point of investment is no longer significant (regression coefficient is –0.045, *t* value is –1.16), but significant with cooperation belief (regression coefficient is 0.903, *t* value is 23.33). Cooperation belief completely mediates the relationship between the horizontal reference point of investment and individual investment. H3B is verified.

**Table 10:** Regression results of intermediary model of horizontal reference point of investment-cooperative belief-individual investment.

	Model (1) Intermediary variable:cooperative belief	Model (2) Dependent variable:individual investment	Model (3) Dependent variable:individual investment
Horizontal reference point (independent variable)	0.811^***^ (48.77)	0.688^***^ (25.05)	−0.045 (−1.16)
Cooperative belief (intermediary variable)			0.903^***^ (23.33)
Constant	2.805^***^ (8.21)	3.956^***^ (7.02)	1.422^***^ (2.94)
Adjusted *R*^2^	0.651	0.330	0.530

*means significant at the level of 10%, **means significant at the level of 5%, ***means significant at the level of 1%. *T* value in parentheses. *N* represents the sample size.

To sum up, the individual investment amount of H1A, H1C, H2A, H2B, and H2C are verified except for H1B, and the relationship between the payoff part and the existence of leaders and the transformation of investment types is unstable; H3A, H3B, H4A, and H4B are verified. One of the remarkable characteristics of the public goods experiment is the vulnerability of cooperation, people’s cooperation level gradually decreases with the repetition of the number of periods (Carrillo et al., 2021). Through the introduction of leaders, this study finds that the number of periods is positively correlated with individual investment. The cooperation level does not continue to decline, but shows an upward trend. The investment of only children is lower than that of not-only children in the presence of leaders, which shows the characteristics of “individual rationality” and is basically consistent with the research of Cameron et al. (2013); Previous studies have found that the cooperation level of male in public goods is higher than that of female. Our study found that there is no significant difference in the cooperation level between males and females.

## Conclusion and discussion

When investigating the relationship between leaders and individual cooperative behavior, we divide leaders into three types of investment according to their investment level: low, medium and high. We use the simple public goods experiment without leadership and sequential public goods with leadership to explore the role of leaders and their influence mechanism. The main conclusions and discussions are as follows:

Leaders play two roles in the market, enterprises and social organizations. One is to provide good or bad information about the project to other organization members in the case of asymmetric information. The second is to influence the decisions of other group members by investing before employees. In order to study the demonstration effect of leaders on team cooperation, we use the sequential public goods experiment to verify whether the existence of leaders can improve the cooperation performance of the team. We found that the existence of leaders does not always improve the level of individual cooperation. This conclusion is consistent with the previous research conclusion of leader style (Tong, 2020; Zeng et al., 2020; Bartels et al., 2022). By controlling the investment of leaders, we found that only when the leader’s investment is close to all his capital the employees will improve the level of cooperation and increase the investment. When the leader’s investment amount is about equal to or lower than the average investment amount of employees, employees will have resistance to this, which is easier to reduce their investment amount and adopt a lower cooperation strategy.

We attempted to explain this phenomenon by the change of investment types and transformation, and leader types in our study are divided into three types: low, medium and high according to the amount of investment. Therefore, the transformation of investigation type can also be said to be the change of leader investment. It is found that when the type of leader changes from low to high, the amount of individual investment increases significantly; In addition, when individuals first encounter low type leaders and then gradually move to high type leaders, there are differences in investment between individuals first encounter high type leaders and then encounter low type leaders, because people’s horizontal reference point in decision-making changes. Therefore, it is necessary to pay attention to the situational dependence of individual decision-making behavior in research and practice.

To further explore the mechanism of leader investment influencing employee team cooperation, we have incorporated employees’ personal characteristics into the research to examine the role of employee heterogeneity in leader types and individual investment. Our study found that risk preference and individual investment is a significant positive correlation in the presence of leaders; Horizontal reference point and risk preference play a moderating role in the type of leader and the amount of individual investment; Cooperative belief in social norms has a complete mediating effect in horizontal reference point and individual investment. The horizontal reference point and risk preference all play a positive role among them. Therefore, when the leader type changes from low to high, the higher the horizontal reference point, the more individual investment; the more individual risk preference, the higher the amount of investment. Cooperative belief in social norms plays an important role in individual investment behavior. It can completely mediate the horizontal reference point to affect the level of individual cooperation.

## Limitations and future directions

This research has several limitations. First, when investigating the impact of leader type and change on individual cooperative behavior, we control the leader type and lacks interaction between leaders and individuals. Leaders also have the characteristics of reciprocity and altruism. In future studies, we plan to use real leaders and increase the interaction between leaders and members through communication. Second, we only focused on the impact of leaders on personal investment, but personal investment also impacts leaders’ behavior and decision making. Future research can control employees’ decision-making behavior and investigate its impact on leaders’ decision-making behavior, that is, the behavior change characteristics of leaders when they meet good followers and bad followers. Third, given that our participants were only some college students, whether our findings could be generalized to national universities and even enterprise organizations remains an open question (Li et al., 2020). To provide solid support for the generalizability of our findings, future research should test whether these findings also apply to real business organizations.

## Data availability statement

The original contributions presented in the study are included in the article/supplementary material, further inquiries can be directed to the corresponding author.

## Author contributions

XF and CL conceived the idea of the manuscript and designed the research. recruited subjects, and completed the experiment. XF analyzed the data and wrote the manuscript, whereas CL and JF modified the manuscript. All authors contributed to the article and approved the submitted version.

## Funding

This study was supported by the National Social Science Fund of China (grant numbers: 21BGL238), Cultivation Programmer for Young Backbone Teachers in Colleges and Universities in Henan Province (grant numbers: 2020GGJS087), Humanities and Social Science Research in Colleges and Universities in Henan Province (grant numbers: 2021-ZZJH-076 and 2023-ZDJH-024), Innovation Team of Philosophy and Social Sciences in Colleges and Universities in Henan Province (2019-CXTD-04), and Research and Practice Project of Undergraduate Education and Teaching Reform in Henan University of Technology (JXYJ2021021), social science research topics of Zhengzhou (ZSLX20221068), and Doctoral Research Fund of Zhengzhou University of light industry (2018BSJJ067).

## Conflict of interest

The authors declare that this research was conducted in the absence of any commercial or financial relationships that could be construed as a potential conflict of interest.

## Publisher’s note

All claims expressed in this article are solely those of the authors and do not necessarily represent those of their affiliated organizations, or those of the publisher, the editors and the reviewers. Any product that may be evaluated in this article, or claim that may be made by its manufacturer, is not guaranteed or endorsed by the publisher.

## Appendix

The payoff of function member i in t period is:

$$\begin{array}{l} \pi_{\mathrm{it}}=x_{\mathrm{it}}+\alpha_{t}\left(\overset{n}{\underset{j=1}{\sum }}g_{{}_{jt}}\right)=e-g_{{}_{\mathrm{it}}}+\alpha_{t}\left(\overset{n}{\underset{j=1}{\sum }}g_{{}_{jt}}\right)=\\ \;e-\left(1-\alpha_{t}\right)g_{{}_{\mathrm{it}}}+\alpha_{t}\left(\overset{n}{\underset{j=1,j\neq i}{\sum }}g_{{}_{jt}}\right)\text{,} \end{array}$$

if the participant is a “rational person” in the sense of economics, he will maximize his own interests. According to the constraint condition of $0\leq \alpha_{t}<1$, the participant will choose to invest $g_{\mathrm{it}}=0$ in the collective project, his final payoff is $e+\alpha_{t}\left(\overset{n}{\underset{j=1,j\neq i}{\sum }}g_{jt}\right)$. However, in terms of maximizing the overall social income, according to the payoff of function member i in t period

$$\pi_{\mathrm{it}}=x_{{}_{\mathrm{it}}}+\alpha_{t}\left(\overset{n}{\underset{j=1}{\sum }}g_{{}_{jt}}\right)=e-g_{{}_{\mathrm{it}}}+\alpha_{t}\left(\overset{n}{\underset{j=1}{\sum }}g_{{}_{jt}}\right)$$,

and $n\alpha_{t}>1$, the participant will choose to invest $g_{\mathrm{it}}=e$ in the collective project. His final payoff is $\alpha_{t}\left(\overset{n}{\underset{j=1}{\sum }}g_{jt}\right)$, and the overall social income is $n\alpha_{t}$ $\left(\overset{n}{\underset{j=1}{\sum }}g_{jt}\right)$.
